# Supplementary material for: Learning curve and surgical outcome of robotic assisted colorectal surgery with ERAS program
Source: Sci Rep. 2022 Nov 29;12:20566. doi: 10.1038/s41598-022-24665-w (PMC9709162; doi:10.1038/s41598-022-24665-w)
Supplement: Supplementary file 2 — Supplementary Information 2. [file 41598_2022_24665_MOESM2_ESM.zip › RAL Raw data-Ñ[▒K/057/OP N_ñw╝╨░O▒Kñσ.pdf]

W55 / W55

臺中榮民總醫院
手術室護理記錄

[redacted]

基本資料

- 室別： 開刀房-17室，手術科別： CRS，手術類別： 常規，日期： 2021/01/27
- 進入時間： 1620 ； 麻醉開始時間： 1635 ； Time Out時間： 1805 ； 劃刀時間： 1806 ； 手術結束時間： 0140 送出時間： 0200 ；
- 麻醉方式： GE
  診斷：Malignant tumor of D-colon
  手術名稱：Robot Assisted Colectomy(L)
  手術局部用藥：nil
  術前預防性抗生素：
  - 抗生素名稱一： Veterin 2GM IVA/由麻醉護理師給予
  - 時間： 1750
  - 上次簽章: 陳盈妤
  ，追加預防性抗生素：
  - 抗生素名稱一： Veterin 1GM IVD由麻醉護理師執行
  - 時間： 2150
  - 上次簽章: 邱任宏
- 手術醫師： 林俊余，林陳彥，陳凱祥，張廷羽
- 麻醉醫師： VS 謝昀叡
-
  - 刷手護士A ： 陳映庭，時間： 1620，刷手護士B ： 陳盈妤，時間： 1620，接替刷手一： 呂昭蓉，時間： 1730，接替刷手二： 蘇郁安，時間： 0030
  - 流動護士A ： 呂昭蓉，時間： 1620，接替流動一： 陳盈妤，時間： 1700，接替流動二： 陳映庭，時間： 1800
- 更多接替及流動護士： 接替流動三： 邱任宏，時間： 2000，接替流動四： 林宜旻，時間： 2300

皮膚狀況

A-V Shunt： 無
術前： 完整
記錄者： 上次簽章: 呂昭蓉
術後： 完整
記錄者： 林宜旻

手術準備

手術臥式 單一手術臥式

-
  - 手術臥式： 截石臥式，NIL
  - 使用附件： 溫毯 38 ℃，頭圈，其它： Slicon padx2 ` lateral supportx2
  - 皮膚準備： 10%Aq Betadine，Hibiscrub，2% Chlorhexidine，其它： NIL
  - 電燒： 使用電燒: 電極片位置： 右大腿，雙極電燒，記錄者： 上次簽章: 陳盈妤

止血帶T： 無

植入物

- 植入物名稱/號碼/數量/部位：Davunci staper 45-mm 4.3mm/2釘/colon
  Hyalo barrier gel 10ml/壹/腹部

，記錄者： 上次簽章: 林宜旻

標本送檢

- 細菌培養 無
- 細胞學 無
- 冰凍切片 無
- 病理標本
  D-S colon

組織名稱一：
,
組織名稱二： Distal cutend
,
組織名稱三： IMA root
, 更多組織名稱
  - 組織名稱四： IMV root

- 截肢 無

標本交付 標本： 肆 袋，標本： 零 盒，標本： 零 瓶，細胞學： 零 管，Culture Tube： 零 管，被交付醫師： R 張廷羽，

- 記錄者： 林宜旻
- 時間： 0155

傷口術後數料

傷口數料： 傷口數料： 無

- 請於圖上標示部位：

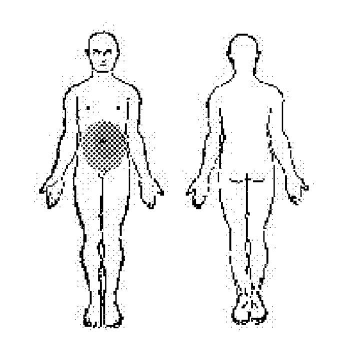

腹腔鏡伍切口、左腹壹切口

- 傷口數料： Mepore

導管

導管	天數	導管資訊	插入日期	插入時間	更換日期	拔除日期	拔除時間	拔除原因	預計拔除/更換	預計拔除/更換日期
A-line (1)	2	號碼:20G ; 部位:左手;	2021/01/27	1720						
Foley(1)	2	號碼:16 Fr.; 部位:膀胱; 註記:Ballon 10ml;	2021/01/27	1720						
Double Lumen CVC(1)	2	號碼:8Fr. ; 部位:; 象限:RU; 註記:fix 15cm;	2021/01/27	1715						

用物計數

- 手術中出血量： 200 c.c.
- 手術中尿量： 1300 c.c.
-
  - 其它： 無
  - NIL

用物計數： 多切口同時縫合

- 1.器械： 正確，2.縫針： 正確，3.刀片： 正確，4.紗布： 正確，5.腹部墊： 未使用，6.紗球： 未使用，7.PEANUTS ： 未使用，8.紗條： 正確，9.絲帶： 未使用，10.針頭： 未使用，11.Vessel loop： 未使用，12.Boots： 未使用，13.Jelco： 未使用，14.棉條： 未使用，
  - 第一次：
    - 刷手護士 ： 蘇郁安
    - 流動護士： 上次簽章: 林宜旻
    - 時間： 0100

  ,
  - 第二次：
    - 刷手護士 ： 蘇郁安
    - 流動護士： 上次簽章: 林宜旻

- 時間： 0110

,

- 第三次：
  - 刷手護士 ： 蘇郁安
  - 流動護士： 上次簽章: 林宜旻
  - 時間： 0125

備註

備註：XI Davinci scope 0+30度NO.1

- 記錄者： 林宜旻 上次簽章: 林宜旻
- 時間： 0200

填寫護理人員 林宜旻

正式摘要

W55 / W55

doctorRank:B
